# Impacts of Seasonal Malaria Chemoprevention on Malaria Burden among under Five-Year-Old Children in Borno State, Nigeria

**DOI:** 10.1155/2020/9372457

**Published:** 2020-07-01

**Authors:** J. P. Ambe, S. T. Balogun, M. B. Waziri, I. N. Nglass, A. Saddiq

**Affiliations:** ^1^Department of Paediatrics, Faculty of Clinical Sciences, College of Medical Sciences, University of Maiduguri, Maiduguri, Nigeria; ^2^Department of Clinical Pharmacology and Therapeutics, Faculty of Basic Clinical Sciences, College of Medical Sciences, University of Maiduguri, Maiduguri, Nigeria; ^3^State Malaria Elimination Programme, Ministry of Health, Maiduguri, Borno State, Nigeria; ^4^World Health Organization, North East Zone, Nigeria

## Abstract

Malaria disproportionately affects all ages with a high burden among children below five years. Thus, control measures are deployed including Seasonal Malaria Chemoprevention (SMC). The present study assessed the impacts of SMC on malaria burden among subjects aged 3–59 months in Borno State, Nigeria. Twenty (20) clusters were randomly selected from accessible 16 Local Government Areas (LGAs) of Borno State, Nigeria, and SMC was deployed in 10 of the clusters by administering a full dose of amodiaquine plus sulfadoxine-pyrimethamine at monthly intervals for 4 months consecutively. Three hundred and ninety-nine children were enrolled in the study. A structured questionnaire was used to obtain demographic and malaria-related data. Thick blood smear, thin blood smear, and capillary sample were collected two weeks after the 4^th^ cycle of SMC. The prevalence of malaria and anaemia was determined among the subjects and for the clusters. The proportions of the female (46.4%; 185/399) and male (53.6%; 214/399) subjects were similar (*p* > 0.05) with subjects aged 24–47 months (35.8%; 143/399) accounting for the highest proportion (*p* < 0.05). Malaria prevalence was 10.3% (41/399) and was higher among non-SMC subjects (15.9%; 31/195) than among SMC subjects (4.9%; 10/204) (*p* < 0.05, df = 1, *χ*^2^ = 10.8). Malaria prevalence was higher in non-SMC clusters (80.0%; 8/10) than in SMC clusters (30.0%; 3/10) (*p* < 0.05, df = 1, *χ*^2^ = 40.5). The mean haematocrit of the 399 subjects was 34.0 ± 5.3% with an anaemia prevalence of 18.1% (72/399). The mean haematocrit was higher among SMC subjects (35.4 ± 5.0% vs. 33.1 ± 4.2%; *p* < 0.05) while anaemia prevalence was higher among non-SMC subjects (21.5% vs. 14.6%; *p* < 0.05, df = 1, *χ*^2^ = 2.8). Of the SMC subjects, 4.9% reported adverse drug reactions. SMC is safe and significantly reduced malaria burden among children in Borno State, and thus, the measure could be deployed in the state for effective malaria control.

## 1. Introduction

Malaria is a febrile protozoan infection of global public health concern with most burden experience in sub-Saharan Africa and Southeast Asia where the disease is endemic. Despite the widely reported reduction in the global malaria burden, malaria continues to constitute a significant challenge [1] with 216 million acute cases and 445,000 malaria deaths reported worldwide in 2016 [2, 3]. A significant proportion of the reported malaria morbidity (90%) and mortality (91%) occurs in sub-Saharan Africa [3] where children below 5 years and pregnant women are at the highest risk of the infection [4]. In addition, 80% of the global malaria burden reported in only 15 countries out of the 91 countries with indigenous malaria cases. All the 15 countries except India are in sub-Saharan Africa, and Nigeria ranked as one of the high malaria burdened countries [3].

World Health Organization (WHO) recommends several malaria preventive measures which include prompt diagnosis and treatment; chemoprevention including Seasonal Malaria Chemoprevention (SMC), intermittent preventive treatments in pregnancy (IPTp) and infancy (IPTi); integrated vector control including long-lasting insecticidal nets (LLINs) and indoor residual spraying (IRS); and larval source management including environmental management and modification and biolarvicide use [5–9]. SMC is the complete treatment course with a fixed-dose combination of amodiaquine and sulfadoxine-pyrimethamine given at monthly intervals to children aged 3–59 months, beginning at the start of the transmission season [7, 8]. The SMC is recommended for the prevention of falciparum malaria in high seasonal transmission areas of sub-Saharan Africa. The delivery of the medication is primarily door-to-door in most countries and this has significantly improved coverage areas. Nigeria is one of the beneficiaries of the SMC scaled-up programme with 6.3 million doses of the drugs administered to children across the states between 2012 and 2016 [3]. SMC is routinely used in some Sahel states in northern Nigeria. It has shown to be an effective malaria control measure; however, cautions are required in regions (Nigeria inclusive) where resistance to amodiaquine and sulfadoxine-pyrimethamine is an issue of concern. Thus, the need for periodic monitoring of the effectiveness of the SMC drugs in these regions.

Borno State, Nigeria, is the largest and most populous state in northeast Nigeria with a population of over 4 million as at 2006 distributed across the 27 Local Government Areas (LGAs) of the state [10]. However, the population distribution of the state has been significantly distorted with over 2 million internally displaced persons (IDPs) due to the protracted armed conflict in the region. The health facilities are overstretched due to the overwhelming number of IDPs in most accessible towns in the state. The crisis in the region has complicated the burden of malaria and other health conditions that prompted governments and nongovernmental organizations to provide number of intervention programmes [11]. Thus, the present study assessed the impacts of SMC on malaria burden among subjects aged 3–59 months in Borno State, Nigeria.

## 2. Materials and Methods

### 2.1. Study Area and Population

**Figure 1:**
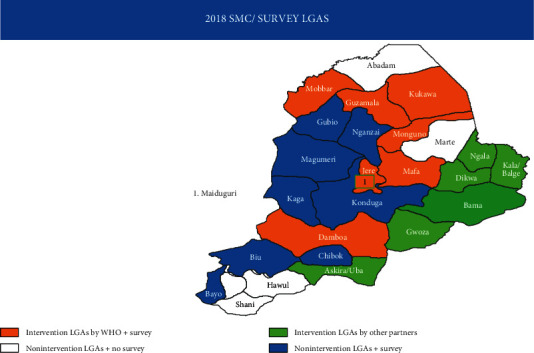
Map showing the 27 Local Government Areas of Borno State.

The study was conducted in Borno State, Nigeria, located on latitude 11^o^05′N and longitude 13°05′E with a land area of 70,898 km^2^, a population of over 4 million, and a population density of 58.8 persons/km^2^ as at 2006 [10]. The state comprises 27 LGAs (Figure 1) and shares internal borders with Adamawa, Yobe, and Gombe states and international borders with Republics of Chad, Cameroon, and Niger. The inhabitants of the state are predominantly Kanuri; other ethnic groups are Babur, Bura, Marghi, Shuwa, Hausa, Fulani, Yoruba, and Igbo, among others. The major economic activities of the people include farming, livestock keeping, trading, fishing, artisanship, and civil service. The climate is characterized by cold-dry (October-February), hot (March-May/June), and rainy seasons (June or July-September) [12]. The average annual rainfall is 562 mm, the average annual climatic temperature is 26.9°C [13], and the relative humidity is 15% (March) to 72% (August) [14]. There is poor environmental sanitation [15] which could promote malaria burden.Malaria transmission peaks during rainy season thereby making the transmission seasonal in Borno State [16] with recently reported prevalence of 78.5% among under five years and 43.2–84.2% during the rainy season [17].

### 2.2. Ethical Consideration

The ethical approvals for the study were obtained from the Ministry of Health, Borno State, and the Research Ethics Committee, World Health Organization. In addition, written informed consent was obtained from the parents/guidance of all enrolled subjects and where necessary permission was sought from the community leaders in various clusters. The study was conducted according to the best clinical and laboratory practices.

### 2.3. Study Design and Selection of Clusters

The study was a cross-sectional study aimed at assessing malaria burden in two cohorts of subjects, viz.: (i) subjects who received SMC regarded in this article as SMC subjects and (ii) subjects who did not receive SMC regarded in this article as non-SMC subjects. Twenty clusters were randomly selected across the 16 accessible LGAs, and of these, 10 clusters were again randomly selected to receive SMC intervention.

### 2.4. Seasonal Malaria Chemoprevention Intervention

Between 14 July and 12 November 2018, all children <5 years old in the 10 clusters randomly selected from 7 LGAs to participate in SMC intervention were given the SMC drugs. Prior to the administration of the drugs, the children were screened for malaria using microscopy and/or rapid diagnostic techniques [18]. Children without malaria parasitaemia were given 4 doses of amodiaquine and sulfadoxine-pyrimethamine with at least one-month interval between two successive doses as described by WHO [7, 8], while children with malaria parasitaemia were first treated with artemether-lumefantrine [19] and then given SMC regimen following confirmation of parasitaemia clearance.

### 2.5. Subject Enrolment and Sample Collection

Trained fieldworkers comprising laboratory technologists/technicians, medical record officers, and nurses/counsellors were deployed to the 20 clusters on 26^th^ and 27^th^ of November 2018 for subject enrolment and data collection (two weeks after 4^th^ cycle of SMC). In each of the 20 clusters, 20 households were selected based on a preexisting sample frame being used for National Immunization Programme in Nigeria [20]. A child aged 3–59 months was randomly enrolled from each of the selected households to give a total of 400 children following a written informed consent provided by the parents/guardians. A validated, structured questionnaire was used to obtain demographic and malaria-related data about the enrolled subjects from the parents/guardians. These data included subject age, sex, ownership of LLINs, and fever episodes. Fever was defined as an axillary temperature above 37.5°C [21]. In addition, thick and thin blood smears and capillary samples were collected from finger-pricked blood for the detection of malaria parasites, identification of the species, and haematocrit determination, respectively [22]. All subjects with malaria parasitaemia were treated according to the guidelines for the treatment of uncomplicated malaria [19].

### 2.6. Laboratory Procedures

The thick and thin blood smears were stained with freshly prepared 3% Giemsa stain. The Giemsa-stained thin smears were used for the identification of *Plasmodium* species while the thick smears were used for the estimation of parasite density according to WHO protocol [22]. Smears were declared negative when no parasite was found after viewing 100 hpf by two independent assessors. Then, parasite density was estimated by counting asexual parasites relative to 200 leukocytes and assuming a leukocyte count of 8000 cells/*μ*l blood [22] using the following formula:

(1) $$\begin{array}{c} \text{parasite density}\left(\text{parasites}/\mu \text{l blood}\right)=\frac{\text{number of asexual parasites}}{200\text{ leukocytes}}\times 8000. \end{array}$$

The capillary blood samples were used to determine the haematocrit values for all the enrolled subjects using the microhaematocrit method, and the proportion of the subjects with anaemia, defined as haematocrit <30% [23, 24], was determined.

### 2.7. Statistical Analyses

Data generated from the studies were analyzed using SPSS version 16.0 (SPSS Inc., USA) [25] and presented in text, tables, and figures. Proportions were compared by chi-square test with Yates' or Fisher exact tests, and correlation was assessed by Pearson test while means were compared by analysis of variance (ANOVA) and unpaired *t*-test. Significance was inferred at *p* < 0.05.

## 3. Results

### 3.1. Demographic Characteristics of the Subjects

In all, 1,159 children aged 3–59 months were recorded in the households where the 399 subjects with valid data were enrolled giving an average of approximately 3 children per household.  Table 1 presents the demography of the 399 enrolled subjects. Households with 3 children within the age bracket accounted for the highest proportion of 31.6% (126/399; *p* < 0.05). Subjects aged 24–47 months accounted for the highest proportion (35.8%, 143/399; *p* < 0.05).

### 3.2. Seasonal Malaria Chemoprevention Intervention in Borno State

**Figure 2:**
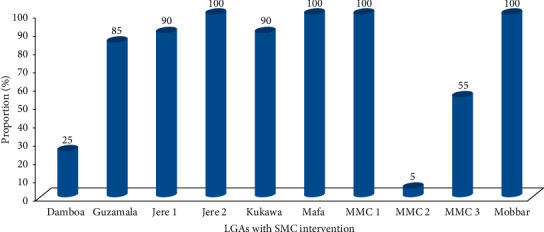
Proportion of 4 cycles of Seasonal Malaria Chemoprevention (SMC) per LGA/cluster.

Overall, 204 subjects received SMC intervention as against expected 200 subjects due to deliberate migration of four subjects from non-SMC clusters to SMC clusters. Approximately 73.0% (149/204) of the subjects received 4 cycles (doses) of SMC drugs as against 5.4% (11/204), 10.8% (22/204), and 10.8% (22/204) who received 3, 2, and 1 dose, respectively (*p* < 0.05), and the full compliance was over 80.0% in seven of the 10 clusters (Figure 2). The reasons provided for noncompliance with the 4 cycles of SMC drugs are travel and terrorists attack in the clusters. Evaluation of the safety of the drugs indicated that only 4.9% (10/204) of the subjects who received the intervention manifested adverse drug reactions which included itching (*n* = 4), rashes (*n* = 3), and vomiting (*n* = 3). Nine of the ten reported cases of the reactions were recorded among subjects enrolled at Guzamala and Kukawa clusters.

### 3.3. Malaria Burden among Subjects Aged 3–59 Months in Borno State

The malaria burden among subjects aged 3–59 months in Borno State is presented in Table 2. Briefly, malaria prevalence among the subjects was 10.3% (41/399) and was higher among non-SMC subjects (15.9%; 31/195) than among SMC subjects (4.9%; 10/204) (*p* < 0.05, df = 1, *χ*^2^ = 10.8). Four of the ten parasitaemic SMC subjects received only one cycle of SMC. The other 6 subjects received 4 cycles and they were enrolled at Mafa (*n* = 2) and Mobbar (*n* = 4) clusters and three of them had a high parasite density of 12,680, 48,600, and 97,600 parasites/*µ*l blood. In addition, the prevalence was highest in Bayo cluster with 55.0% (11/20) and lowest in Kaga cluster with 5.0% (1/20) (*p* < 0.05, df = 19, *χ*^2^ = 96.4). Similarly, cluster-based prevalence indicated higher proportion of non-SMC clusters (80.0%; 8/10) recorded malaria during the study period than SMC clusters (30.0%; 3/10) (*p* < 0.05, df = 1, *χ*^2^ = 40.5). Further analysis showed that children with malaria parasitaemia had a higher probability of developing anaemia (Table 3). It is worthy to note that all the 41 cases of malaria recorded were due to *P. falciparum*.

### 3.4. Anaemia Burden among Subjects Aged 3–59 Months in Borno State

The mean haematocrit of the 399 subjects was 34.0 ± 5.3% (95% CI: 33.5–34.6%) and was similar among the various age groups (*p* > 0.05) and both sexes (*p* > 0.05). However, it was lower among subjects with malaria parasitaemia (*p* < 0.05) and higher among SMC subjects than among non-SMC subjects (*p* < 0.05). Similarly, subjects enrolled at Guzamala cluster (42.5 ± 4.0%) had a significantly higher mean haematocrit (*p* < 0.05) (Table 4). Furthermore, 72 of the 399 subjects had haematocrit below 30% giving anaemia prevalence of 18.1%. Prevalence of anaemia was more among non-SMC subjects (*p* < 0.05, df = 1, *χ*^2^ = 2.8) and subjects enrolled at Biu cluster (*p* < 0.05, df = 19, *χ*^2^ = 65.3) (Table 4).

### 3.5. Fever Episodes among Subjects Aged 3–59 Months in Borno State

**Figure 3:**
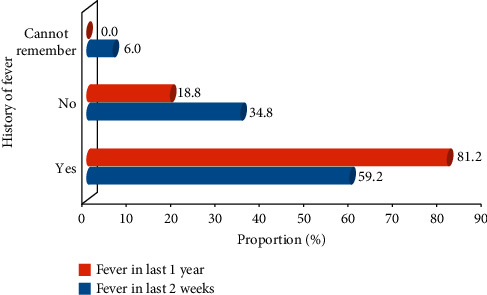
History of fever episodes among the children.

**Figure 4:**
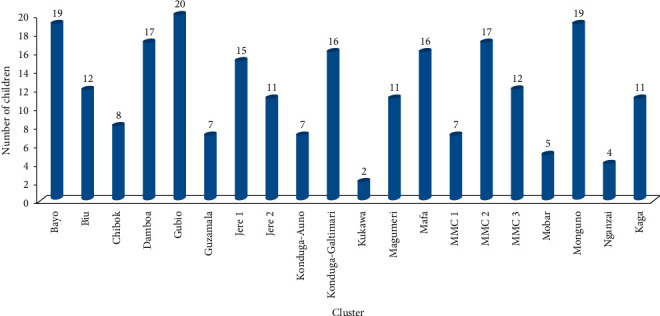
Cluster distribution of children with fever in the last two weeks.

In the present study, the number of fever episodes in the last two weeks (Fever_2wks_) and the last one year (Fever_1yr_) preceding the enrolment and after SMC intervention (Fever_smc_) was investigated among the subjects. The prevalence of Fever_2wks_ and Fever_1yr_ among the subjects was 59.1% (236/399) and 81.2% (324/399), respectively (Figure 3). Fever_2wks_ was higher (*p* < 0.05) among non-SMC subjects (70.2%, 127/181) than among SMC subjects (53.4%, 109/204).  Figure 4 presents the distribution of Fever_2wks_ across the 20 clusters with Gubio cluster having the highest mode of 20. Fever_smc_ (13.7%) among SMC subjects was lower than Fever_2wks_ (59.2%) among all the 399 subjects.

### 3.6. Ownership of Long-Lasting Insecticidal Nets

Ownership of LLINs was assessed in Borno State among the caregivers of subjects. Most of the caregivers (86.0%, 343/399) reportedly owned bed nets as against 56 caregivers (14.0%) who do not have bed nets. These nets are insecticide-treated as reported by 253 (73.8%) of the caregivers who owned bed nets. Approximately two-thirds (249/399) of the respondents reported that their children always sleep under nets which are mainly acquired free from the government (48.6%, 194/399).

## 4. Discussion

Ensuring universal access to malaria prevention, diagnosis, and treatment is the first pillar of the global technical strategy for malaria reduction by 2023 [26]. Antimalarial chemotherapy remains the cornerstone of malaria prevention evident by being an integral component of various preventive measures such as IPTp [5], IPTi [6], and SMC [7, 8]. Previous studies using controlled clinical trials and routine control programmes have demonstrated the efficacy of SMC as an effective malaria control measure [27, 28]. However, drug resistance could hinder the success of the strategy especially in areas where resistance to amodiaquine and sulfadoxine-pyrimethamine is high. Thus, the present study assessed the impacts of SMC on the malaria burden among subjects aged 3–59 months in Borno State, Nigeria.

Compliance with the 4 cycles of SMC was relatively impressive among the clusters where SMC was deployed and this is in accordance with previous studies that have reported high compliance rate with SMC [29, 30]. However, the influence of about one-quartile of the subjects who did not complete the 4 cycles should not be ignored, especially those that received only one or two cycles. This could result in fluctuation of plasma drug level below the therapeutic level during the malaria transmission season, hence predisposing the subjects to the infection and possible emergence of resistance [29]. The 4 cycles of SMC were recommended to maintain therapeutic plasma drug level throughout the 4-month malaria transmission in areas where malaria transmission is seasonal [7, 8]. Borno State, Nigeria, has been the hub of insecurity for about a decade [31] and this explains some of the reasons provided for missing the SMC cycles. Coldiron and others [29] have previously identified insecurity as one of the SMC challenges. Thus, the governments and other stakeholders must intensify efforts to end the crisis in northeast Nigeria so as to ensure effective malaria control in the region. The SMC subjects demonstrated good tolerability of the drugs with only few of the subjects manifesting mild adverse effects such as itching, rashes, and vomiting. This observation is in accordance with previous studies that have demonstrated the safety of amodiaquine and sulfadoxine-pyrimethamine [7, 8, 28].

To the best of our knowledge, this is the first study in recent times that examined the malaria burden among subjects below 5 years across the state. Similarly, this is the first of its kind to examine the impact of SMC on malaria burden in the state. Previous studies done in the state were mostly restricted to Maiduguri, the state capital [17, 32, 33], mainly due to insecurity in most other parts of the state. In this study, the malaria burden was about threefold higher among non-SMC subjects than among SMC subjects. Similarly, non-SMC clusters recorded malaria transmission among subjects aged 3–59 months about threefold more than SMC clusters. These are indications that administered SMC drugs provided a stable therapeutic plasma level that prevented the establishment of erythrocytic stages of malaria parasites even when the subjects were exposed to the infection. This is in agreement with previous reports that SMC is effective in the prevention of malaria [28, 29, 34]. The fact that six subjects who received complete SMC cycles had malaria during the study period is an issue of epidemiological and pharmacological concerns. This may point to the circulation of strains of *P. falciparum* resistant to amodiaquine and sulfadoxine-pyrimethamine in the state, especially in Mafa and Mobbar LGAs where all the six subjects were enrolled. Balogun and others [33, 35] have reported resistance to antimalarial drugs among *P. falciparum* isolated in the state. This necessitates the need for robust assessment of amodiaquine and sulfadoxine-pyrimethamine efficacy in the region prior to routine deployment of SMC for malaria control in the state.

Anaemia burden is routinely used as an indicator of malaria burden especially among under 5-year-old children [20]. In the present study, the impacts of SMC on anaemia burden were investigated among SMC subjects/clusters and non-SMC subjects/clusters. The burden of anaemia was twofold more among non-SMC subjects than among SMC subjects, and the haematocrit levels of SMC subjects were significantly higher than those of non-SMC subjects. Several anaemia-inducing factors that affect haematocrit levels have been identified including nutritional status, genetic disorders, and infections [36, 37]. The disparity in the anaemia burden observed in the two cohorts of the subjects could be mainly attributed to variation in the malaria burden among the two cohorts. Hence, it could be opined that SMC reduced malaria burden which in turn resulted in reduction in anaemia burden among the subjects that received SMC. This is similar to studies that previously reported that SMC intervention reduced the burden of malaria indicators including anaemia [28, 29, 34]. However, it is noteworthy to state that one out of two subjects (50%) enrolled at Biu cluster had anaemia despite that only one out of ten subjects (10%) had malaria parasitaemia. This may indicate other anaemia-inducing factors among Biu subjects that may require further investigation.

Furthermore, episodes of fever were investigated as malaria indicator in children [10]. The episodes of Fever_2wks_ and Fever_1yr_ were more among non-SMC subjects than among SMC subjects. This finding could point to the fact that SMC intervention significantly reduced the chances of children below 5 years to develop fever. This is in accordance with reports that adequately deployed SMC reduced the chance of malaria transmission and subsequent fever development in children [28, 29]. LLINs are one of the effective measures to protect against malaria transmission especially in sub-Saharan Africa [9, 38]; thus, ownership of LLINs was also assessed in the present study. Majority of the households sampled in this study owned LLINs with 647 LLINs declared in 399 households having 1,159 children below 5 years. This appears adequate if the nets are dedicated to these children. Thus, effective use of the nets might have contributed to the reduced burden of malaria observed among SMC cohorts. However, the similarity between LLINs ownership among SMC and non-SMC groups suggests otherwise. In addition, the fact that older members of the households may jointly use the nets demands for additional preventive measures. Thus, SMC intervention may provide the required additional control measures especially during the malaria transmission season as the previous study has shown synergy between two malaria preventive measures [39].

## 5. Conclusion

The present study indicated that SMC is effective and safe when used for malaria prevention among children in Borno State, Nigeria. It effectively reduced malaria burden by threefold among children, improved their haematocrit values by twofold, and decreased incidence of fever episodes by fourfold. Thus, SMC should be considered as an additional malaria prevention measure in the state especially in areas that are accessible following improved security in the region.

## Figures and Tables

**Table 1:** Demography of the children enrolled in the study.

Variable	Number (%)	*p* value
Number of households sampled	399 (100.0)	—

Number of children enrolled^*∗*^	399 (100.0)	—

Number of children aged 3–59 months (per household)		
1	59 (14.8)	<0.05
2	114 (28.6)	
3	126 (31.6)	
4	62 (15.5)	
≥5	38 (9.5)	

Age group (months)		
<6	21 (5.3)	<0.05
6–11	56 (14.0)	
12–23	107 (26.8)	
24–47	143 (35.8)	
48–59	72 (18.1)	

Sex		
Female	185 (46.4)	>0.05
Male	214 (53.6)	

^*∗*^One child per household.

**Table 2:** Prevalence of malaria among the children.

Cluster	SMC intervention	Number of children		Malaria prevalence (%)^*∗*^	GMPD (parasite/*µ*l)
		Recruited	With malaria		
Bayo	No	20	11	55.0	2,099
Biu	No	20	2	10.0	51
Chibok	No	20	2	10.0	202
Damboa	Yes	20	4	20.0	355
Gubio	No	20	4	20.0	1,051
Guzamala	Yes	20	0	0.0	—
Jere 1	Yes	20	0	0.0	—
Jere 2	Yes	20	0	0.0	—
Konduga-Auno	No	20	3	15.0	3,471
Konduga-Galtimari	No	20	5	25.0	597
Kukawa	Yes	20	0	0.0	—
Magumeri	No	20	3	15.0	1,769
Mafa	Yes	20	4	20.0	2,554
MMC 1	Yes	19	0	0.0	—
MMC 2	Yes	20	0	0.0	—
MMC 3	Yes	20	0	0.0	—
Mobbar	Yes	20	2	10.0	9,011
Monguno	No	20	0	0.0	—
Nganzai	No	20	0	0.0	—
Kaga	No	20	1	5.0	240
Total	—	399	41	10.3	1,119

GMPD: geometric mean parasite density. ^*∗*^*p* < 0.05. SMC: Seasonal Malaria Chemoprevention.

**Table 3:** Factors influencing malaria parasitaemia among the children.

Variable	Number recruited	Malaria parasitaemia		*p* value
		Frequency	Percentage (%)	
Anaemia (haematocrit <30%)				
Yes	72	18	25.0	<0.05
No	327	23	7.0	

Age (months)				
<6	21	1	4.8	>0.05
6–11	56	5	8.9	
12–23	101	9	8.4	
24–47	143	1	11.9	
48–59	72	9	12.25	

Sex				
Female	185	17	9.2	>0.05
Male	214	24	11.2	

SMC intervention				
Yes	204	10	4.9	<0.05
No	195	31	15.9	

SMC: Seasonal Malaria Chemoprevention.

**Table 4:** The burden of anaemia among the children.

Variable	Haematocrit (%)	Subjects with anaemia		^*∗*^ *p* value
		Number (%)	Haematocrit (%)	
Age (months)				
<6	33.9 ± 4.8	5 (23.8)	27.6 ± 2.1	>0.05
6–11	33.1 ± 4.3	12 (21.4)	28.0 ± 0.7	
12–23	34.3 ± 4.1	15 (14.0)	27.4 ± 1.7	
24–47	34.1 ± 5.1	33 (23.1)	27.7 ± 1.2	
48–59	35.4 ± 5.2	7 (9.7)	27.1 ± 1.1	

Cluster				
Bayo	31.4 ± 3.9	7 (35.0)	27.0 ± 1.7	<0.05
Biu	29.9 ± 2.4	10 (50.0)	27.9 ± 1.0	
Chibok	34.8 ± 3.4	1 (5.0)	29.0	
Damboa	31.9 ± 4.7	7 (35.0)	27.3 ± 0.8	
Gubio	30.8 ± 4.2	9 (45.0)	27.3 ± 2.0	
Guzamala	42.5 ± 4.0	0 (0.0)	—	
Jere 1	35.7 ± 3.5	2 (10.0)	28.5 ± 0.7	
Jere 2	32.8 ± 4.0	6 (30.0)	28.2 ± 0.8	
Konduga-Auno	35.1 ± 3.6	1 (5.0)	29.0	
Konduga-Galtimari	34.8 ± 4.1	2 (10.0)	27.5 ± 0.7	
Kukawa	35.2 ± 4.6	3 (15.0)	28.3 ± 0.6	
Magumeri	33.5 ± 4.0	3 (15.0)	26.7 ± 1.5	
Mafa	31.6 ± 3.7	7 (35.0)	27.7 ± 1.4	
MMC 1	36.6 ± 4.0	0 (0.0)	—	
MMC 2	34.6 ± 3.4	2 (10.0)	28.0 ± 0.7	
MMC 3	34.7 ± 4.0	1 (5.0)	28.0	
Mobbar	38.0 ± 3.9	1 (5.0)	29.0	
Monguno	31.2 ± 4.5	7 (35.0)	27.3 ± 1.4	
Nganzai	35.1 ± 3.7	1 (5.0)	28.0	
Kaga	34.4 ± 4.2	2 (10.0)	27.5 ± 0.7	

Parasitaemia				
Yes	31.0 ± 4.2	18 (43.9)	27.2 ± 1.7	<0.05
No	34.6 ± 4.7	54 (15.1)	27.8 ± 1.1	

Sex				
Female	34.0 ± 4.7	33 (17.8)	27.5 ± 1.6	>0.05
Male	34.4 ± 4.8	39 (18.2)	27.8 ± 1.0	

SMC intervention				
Yes	35.4 ± 5.0	29 (14.6)	27.9 ± 1.0	<0.05
No	33.1 ± 4.2	43 (21.5)	27.5 ± 1.4	

SMC: Seasonal Malaria Chemoprevention. Haematocrit values are presented in mean ± standard deviation. ^*∗*^Compared the prevalence of anaemia.

## Data Availability

The data used to support the finding of the study are available from the corresponding author upon request.
